# Supplementary material for: PML Regulates the Epidermal Differentiation Complex and Skin Morphogenesis during Mouse Embryogenesis
Source: Genes (Basel). 2020 Sep 25;11(10):1130. doi: 10.3390/genes11101130 (PMC7600374; doi:10.3390/genes11101130)
Supplement: Supplementary file 1 [file genes-11-01130-s001.pdf]

## Supplemental figures

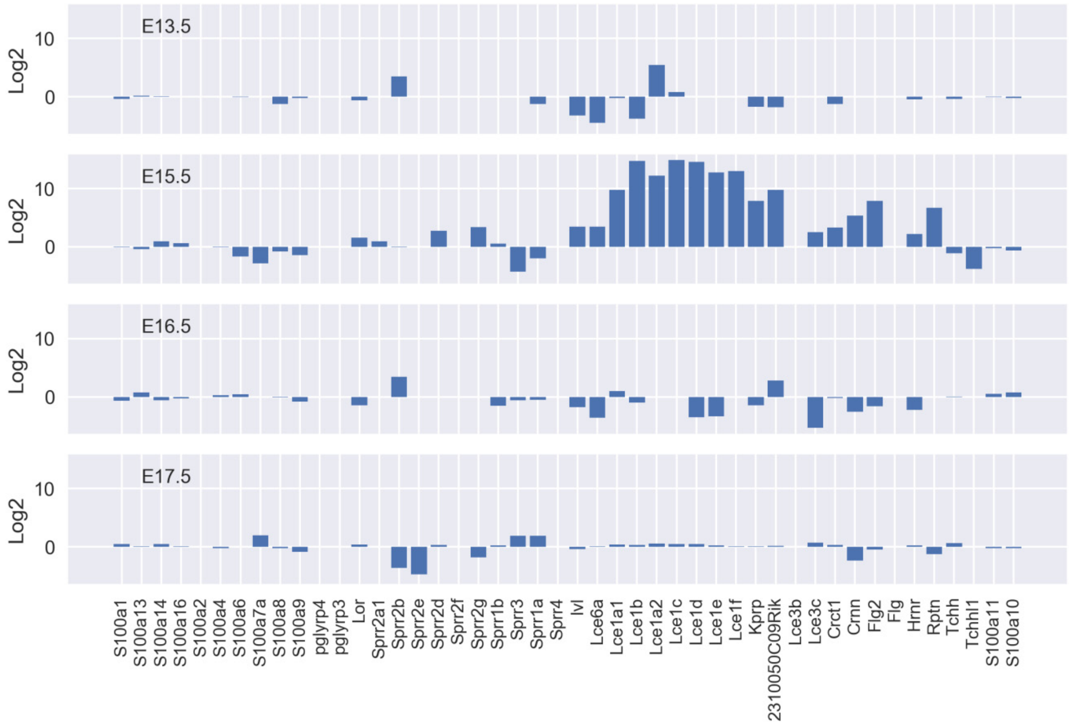


**Figure S1.** Filtered data from RNA-seq analysis showing increased expression from the LCE sub-region of the EDC gene locus at stage E15.5 in the absence of PML.
